# Antifilarial activity of diterpenoids from *Taxodium distichum*

**DOI:** 10.1186/s13071-016-1592-4

**Published:** 2016-05-31

**Authors:** Vikas Kushwaha, Kirti Saxena, Richa Verma, Shiv K. Verma, Deepali Katoch, Neeraj Kumar, Brij Lal, P. Kalpana Murthy, Bikram Singh

**Affiliations:** Division of Parasitology, CSIR-Central Drug Research Institute, New Campus, BS 10/1, Sector 10, Jankipuram Extension, Lucknow, 226 031 India; Natural Product Chemistry and Process Development Division, CSIR-Institute of Himalayan Bioresource Technology, Palampur, 176 061 HP India; Biodiversity Division, CSIR-Institute of Himalayan Bioresource Technology, Palampur, 176 061 HP India; Present Address: USDA, ARS, APDL, BARC-East Bldg 1001, 10300 Baltimore Avenue, Beltsville, MD 20705 USA

**Keywords:** *Taxodium distichum*, *Brugia malayi*, Labdane diterpenoids, *In vitro* assays, Macrofilaricide, Embryostatic, *Mastomys coucha*, *Meriones unguiculatus*, Diethylcarbamazine, Ivermectin

## Abstract

**Background:**

Lymphatic filariasis caused by *Wuchereria bancrofti*, *Brugia malayi* and *B. timori*, is a debilitating disease with an adverse social and economic impact. The infection remains unabated in spite of treatment with existing antifilarial drugs diethylcarbamazine (DEC) and ivermectin which are chiefly microfilaricides. There is therefore, need for macrofilaricides, embryostatic agents and better microfilaricides. In the present study we explored the antifilarial potential of crude extract and its molecular fractions of the plant *Taxodium distichum* using *in vitro* assay systems and rodent models of *B. malayi* infection.

**Methods:**

Ethanolic extract (A001) of aerial parts of *T. distichum* was solvent fractionated and sub-fractionated. Four molecules, 3-Acetoxylabda-8(20), 13-diene-15-oic acid (K001), Beta-sitosterol (K002), labda-8(20),13-diene-15-oic acid (K003) and Metasequoic acid A (K004) were isolated from the fractions and their structure determined by spectroscopic analysis. The extract, subfractions and molecules were evaluated for antifilarial activity against *B. malayi* by 3-(4,5-dimethylthiazol-2-yl)-2,5 diphenyltetrazolium bromide (MTT) reduction and motility assays *in vitro* and in two animal models, *Meriones unguiculatus* and *Mastomys coucha*, harbouring *B. malayi* infection.

**Results:**

A001 was effective in killing microfilariae (mf) and adult worms *in vitro*. The diterpenoid K003 produced 100 % reduction in motility of both mf and adult worms and > 80 % inhibition in MTT reduction potential of adult female worms. In *B. malayi*-*M. unguiculatus* model, A001 killed all the adult worms in > 80 % of infected animals. K003 was embryostatic (> 95 %) in this model. In the *B. malayi*-*M. coucha* model, K003 killed ~54 % of adult worms (macrofilaricidal activity) and rendered > 36 % female worms sterile; it also stopped any further rise in microfilaraemia after day 42 post-initiation of treatment.

**Conclusion:**

Ethanolic extract of aerial parts of the plant *T. distichum* possesses potent antifilarial activity and the active principle was localised to K003 which showed significant macrofilaricidal activity and late suppression of peripheral microfilaraemia and some embryostatic activity. These findings indicate that labdane diterpenoid molecule(s) may provide valuable leads for design and development of new macrofilaricidal agent(s). To the best of our knowledge, this is the first report on antifilarial efficacy of products from the plant *T. distichum*.

## Background

Lymphatic filariasis (LF), caused by the nematode parasites *Wuchereria bancrofti*, *Brugia malayi* and *B. timori*, is one of the neglected tropical diseases and is recognised as the world’s most disabling and disfiguring parasitic disease with an adverse social and economic impact. Around 1.4 billion people in 73 countries worldwide which includes 553 million in India alone, are at risk of the infection [1, 2].

The adult parasites live in the lymphatics and lymph nodes and produce millions of juvenile worms called microfilariae (mf) which remain in the pulmonary vessels during the day and appear in the peripheral blood at night (nocturnal periodicity). On entering mosquitoes (vector) during blood meal, the mf undergo two moults in the vector and develop into 3rd stage infective larvae (L3). The L3 enter human host during the vector’s blood meal and develop into adult worms after two more moults.

Current methods of controlling the transmission of the infection include administration of the microfilaricides diethylcarbamazine (DEC) and ivermectin either alone or in combination with an anthelmintic albendazole *en masse* to people living in areas endemic to the infection. This Mass Drug Administration (MDA) strategy raised hopes for elimination of this disease, but unfortunately, the infection is unabated due to the technical limitations of MDA strategy [3]. Besides, the adverse effects of these antifilarials, the lack of adulticidal activity in these drugs to complement their microfilaricidal activity for a two-pronged attack on the parasite and the fast emerging drug resistance to ivermectin are compounding the situation [4]. Therefore, there is a clear need to develop new antifilarial agent(s) that possess not only microfilaricidal activity but, most importantly, also macrofilaricidal activity and if possible, adult worm sterilising activity as well. In this direction our laboratories have been systematically screening a large number of plants to identify biologically active molecules against lymphatic filarial parasite [5–11].

*Taxodium distichum* (L.) Rich is an ornamental tree commonly known as Bald cypress because of its deciduous character which is unusual in conifers. It is a slow growing and long lived tree [12]. The plant is native to Mississippi valley north to southern Illinois and the coastal plain from Delaware to Mexico [13]. In India, *T. distichum* tree grows in Himachal Pradesh, Uttarakhand and West Bengal. A literature and patent search show that the plant is rich in monoterpenes, diterpenes, sesquiterpenes, flavonoids and their glycosides [14], and there are reports that the plant products show antiviral [14], cytotoxic [15], antitumor [16], anti-oxidant [17], anti-bacterial [17] and antifungal [18] activities. The present study was aimed at determining whether the plant products have any antifilarial activity. To this end, the aerial parts of the plant were selected for investigation. The ethanolic extract, its fractions, sub-fractions and molecules isolated from them were assayed for antifilarial activity using human lymphatic filarial parasite, *B. malayi in vitro* and in animal models of the infection.

## Methods

### Plant material, extraction, fractionation and isolation of molecules and their structure elucidation

Aerial parts of the plant *T. distichum* were collected in February, 2010 from Palampur (located at 32.12°N, 76.53°E, elevation 1220 m above sea level), Himachal Pradesh, India, by Dr. Brij Lal (Biodiversity department of CSIR-Institute of Himalayan Bioresource Technology, Palampur, India). The collection was made in one season only. A specimen has been deposited in the herbarium (voucher no. 1200) of CSIR-Institute of Himalayan Bioresource Technology, Palampur.

Air-dried aerial parts of *T. distichum* (1 kg) were ground to a coarse powder and extracted with ethanol (2000 ml × 3) in a percolator for 24 h. The combined ethanol extract was concentrated in a rotavapor under reduced pressure and lyophilised to get dried extract (60.2 g, A001). The extract was suspended in water and partitioned successively with n-hexane, chloroform and n-butanol (3 × 150 ml) three times each and the fraction left was the aqueous fraction. Each fraction thus obtained was concentrated in a rotavapor under reduced pressure and lyophilised to yield four different fractions designated as F001 (6 g), F002 (3 g), F003 (30 g) and F004 (20 g). All fractions were assayed *in vitro* for antifilarial activity for selecting the most potent one for further fractionation.

**Fig. 1:**
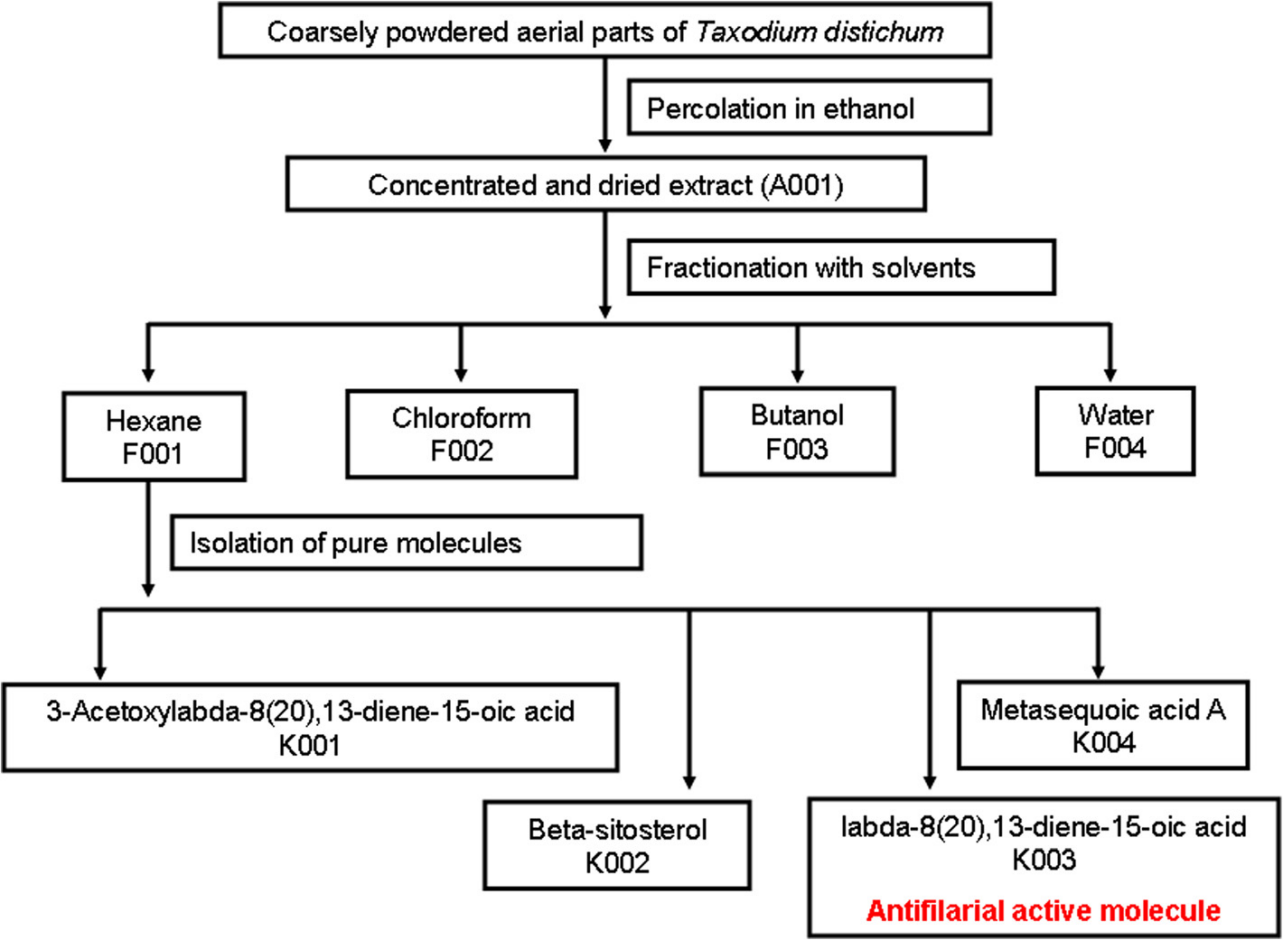
Flowchart of extraction, fractionation and isolation of pure molecules from *Taxodium distichum*

**Fig. 2:**
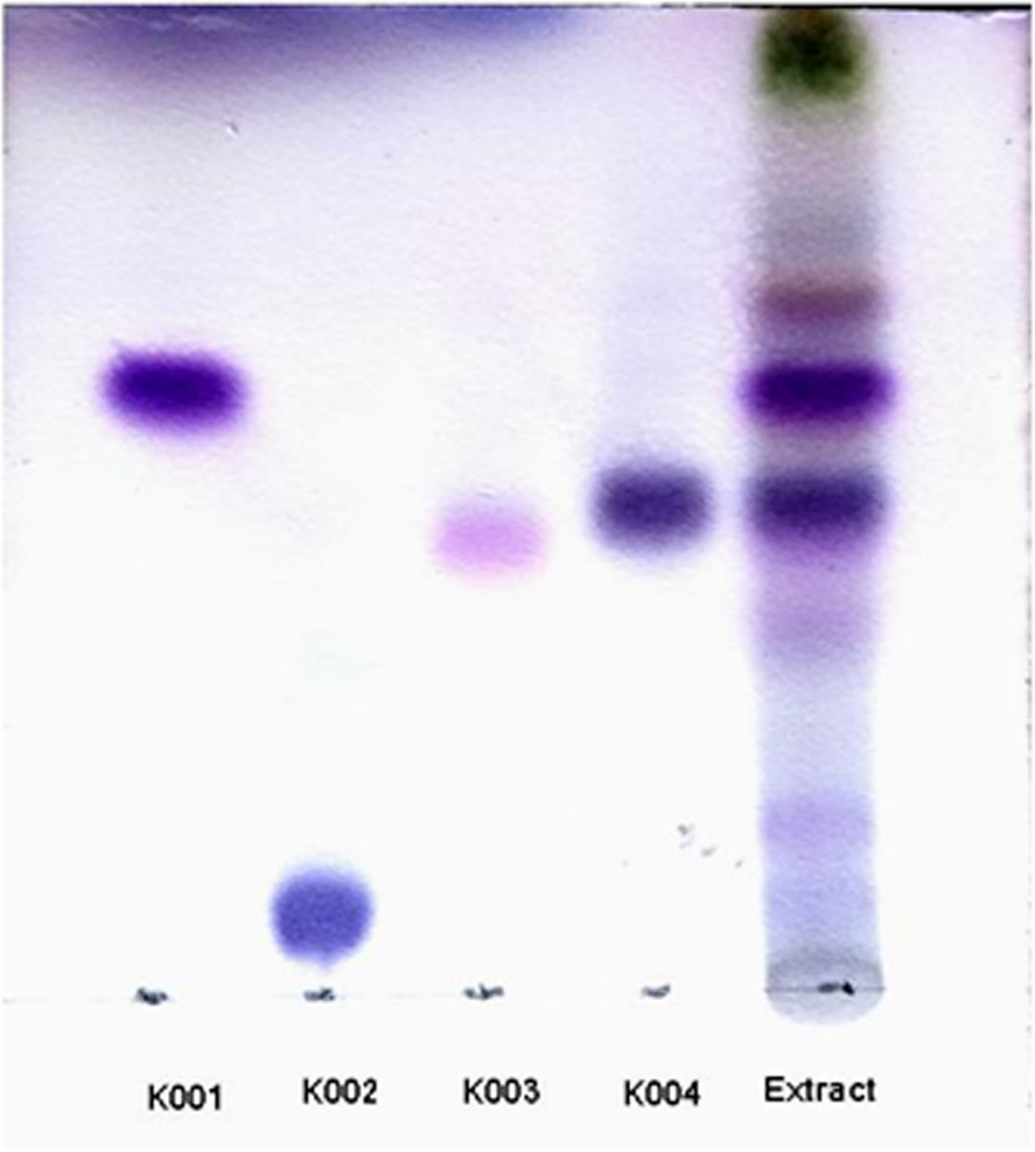
Thin layer chromatography (TLC) of extract and compounds isolated from *Taxodium distichum*

Fraction F001 (*n*-hexane) which was found most effective was subjected to column chromatography over silica gel (100 g, 60–120 mesh, 90 × 6 cm) with gradient of ethyl acetate and *n*-hexane (0.5:10; 0.7:10; 1.25:10; 1.5:10; 1.75:10; 2.0:10; 2.25:10; 2.5:10; 3.0:10; 3.5:10; 4.0:10; 5.0:10; and 7.0:10; v/v). Different fractions of 50 ml volume collected by thin layer chromatography (TLC) monitoring were pooled to six subfractions (SF1-SF6). Compound K001 was isolated after direct crystallisation from SF-4. Further, the subfractions were tested for antifilarial activity. As SF-2 was found to show micro- and macrofilaricidal activity it was subjected to column chromatography separation over silica gel 60–120 mesh (Merck India Ltd.) and eluted with 7 % ethyl acetate in *n*-hexane and fourteen fractions were collected. Compound K002 was isolated by direct crystallisation. Compounds K003 and K004 were purified after repeated column chromatography over RP-18 silica (Merck India Ltd.) using 90 % methanol in water. The flowchart (Fig. 1) shows extraction, fractionation and isolation of molecules. TLC picture of the isolated pure molecules along with the extract was developed on RP-18 F_254_ S TLC plate (Merck India Ltd.) using solvent system Methanol:Water (3:0.05) (Fig. 2). The structures of the isolated compounds were elucidated using 1D, 2D Nuclear Magnetic Resonance (NMR) using Bruker Avance-300 spectrometer, and by mass spectroscopy using Q-TOF mass spectrometer equipped with an ESI source (Micromass, Manchester, UK). The spectroscopic data was compared with the known data available in literature.

### Animals, infection and isolation of parasites

Two rodent species, the southern multimammate mouse *Mastomys coucha* and the jird *Meriones unguiculatus* were obtained from National Laboratory Animal Centre, CSIR-CDRI, Lucknow and used in the present study, these two species are known to be susceptible to *B. malayi* infection. All experiments were conducted in compliance with the Institutional Animal Ethics Committee (IAEC) guidelines for use and handling of the animals (IAEC approval number: IAEC/2010/141). Throughout the study, the animals were housed in climate (23 ± 2 °C; RH: 60 %) and photoperiod (12 h light-dark cycles) controlled animal quarters. They were fed standard rodent “maintenance diet” prepared in-house (QC analysis: carbohydrates 58.30 %, protein 21.10 %, fat 7.20 %, crude fibre 6.60 %, moisture 6.80 %) supplemented with dried shrimps (for *M. coucha*) and had free access to drinking water. Following the guidelines of IAEC, the animals were necropsied using sodium pentothal.

Sub-periodic strain of *B. malayi* was cyclically maintained in *M. coucha* and *M. unguiculatus* through laboratory bred black-eyed filaria susceptible strain of mosquitoes *Aedes aegypti*. Each animal was inoculated with 100 (*M. coucha*) or 200 (*M. unguiculatus*) L3 isolated from experimentally infected *A. aegypti*, through subcutaneous (s.c.) and intraperitoneal (i.p.) routes, respectively [19, 20].

Microfilariae and adult worms were harvested from p.c. of the *M. unguiculatus* harbouring approximately 5–6 month-old *B. malayi* infection. The parasites were washed thoroughly with HBSS (pH 7.2) containing antibiotics (penicillin: 100 U/ml; streptomycin: 100 μg/ml) and used for the present study.

### Evaluation of antifilarial efficacy *in vitro*

Motility and 3-(4,5-dimethylthiazol-2-yl)-2,5 diphenyltetrazolium bromide (MTT; Sigma-Aldrich, USA) reduction assays that indicate viability of the parasites were used [21, 22]. Test samples and ivermectin (purity 95 %; Sigma-Aldrich, USA) were dissolved in dimethyl sulfoxide (DMSO; Sigma-Aldrich, USA); the final concentration of DMSO in the incubation medium Hanks Balanced Salt Solution (HBSS; Sigma-Aldrich, USA) containing 100 U/ml penicillin and 100 μg/ml streptomycin (Sigma-Aldrich, USA), was kept ≤ 0.1 %. Diethylcarbamazine-citrate (DEC-C; purity 100 %; Sigma-Aldrich, USA) solution was prepared in sterile distilled water. HBSS containing DMSO (≤ 0.1 %) alone was used as negative control.

The concentrations of the test agents used were: 500 μg/ml (A001 or its fractions/ subfractions: F001-F004/SF1-SF6), 125 μg/ml (isolated compounds/ molecules: K001-K004), 1000 μM (DEC) and 10/20 μM (ivermectin). *In vitro* efficacy of the test agents and reference drugs was assessed on mf (40–50 mf/100 μl/well) in a 96-well cell culture plate (Nunc, Denmark) and on adult female worms (1 worm/ml/well) in a 48-well plate (Nunc, Denmark). The assays were run in triplicates.

Motility assay using mf and adult worms was carried out as described elsewhere [21–23]. Changes in parasite motility was assessed under a microscope and scored as: 0 = dead (100 %); 1-4 = loss of motility (1 = 75 %; 2 = 50 %; 3 = 25 %; and 4 = no loss of motility). The experiment was repeated twice.

MTT-reduction assay was carried out using adult worms as described elsewhere [22]. The viability of the treated worms was assessed by calculating per cent inhibition in MTT-reduction potential of the treated worms over DMSO treated control parasites [22]. A hundred percent inhibition in motility of female parasites or mf and or ≥ 50 % inhibition in MTT-reduction potential of the treated female parasites were considered to indicate positive antifilarial activity [22].

### Determination of IC_50_ and CC_50_

IC_50_ and CC_50_ of the test agents were determined using the method described elsewhere [9, 23]. For IC_50_, parasite life-cycle stages were incubated with two-fold serial dilutions of the agents (test agents: 7.82–500 μg/ml; DEC-C: 15.63–1000 μM; ivermectin: 0.31–20 μM) in motility and MTT-reduction assays, using triplicate wells of culture plate. The experiments were repeated twice.

In the CC_50_ assay, VERO Cell line C1008 (African green monkey kidney cells) obtained from NLAC, CSIR-CDRI, Lucknow, was incubated with three fold serial dilutions of the test agents and reference drugs (starting from > 20 times conc. of LC_100_ of the agents). The assay was run in replicates in each of the two independent experiments.

Data were transferred to a graphic program (MS Excel) and IC_50_ and CC_50_ were calculated by linear interpolation between the two concentrations above and below 50 % inhibition [24].

Selectivity Index (SI) of the agents was computed by the formula as: SI = CC_50_/IC_50_.

All the *in vitro* tests were repeated twice and Table 1 shows the average values.

**Table 1:** *In vitro* activity of *Taxodium distichum* and the reference drugs ivermectin and diethylcarbamazine (DEC) on adult worms (AW) and microfilariae (Mf) of *Brugia malayi* assessed by motility assay (MA) and MTT reduction assay

Antifilarial agent	LC_100_^a^ (μg/ml) for AW in MA	IC_50_^b^ (μg/ml) for AW in MA	Mean % inhibition in MTT reduction by AW	LC_100_ (μg/ml) for Mf in MA	IC_50_ (μg/ml) for Mf in MA	CC_50_^c^ (μg/ml)	SI for AW in MA	SI for Mf in MA
A001 (crude extract)	15.63	10.00	47.00	3.91	1.95	250	25	128.29
F001	31.25	9.84	79.63	7.83	4.92	210	21.34	42.68
F002	> 125	–	17.00	> 125	88	–	–	–
F003	> 125	–	38.00	> 125	88	–	–	–
F004	> 125	–	42.00	> 125	88	–	–	–
SF1	31.25	12.40	46.23	7.82	< 3.95	250	20.16	> 63.29
SF2	7.82	5.52	78.08	7.82	< 3.95	430	77.89	> 108.86
SF3	> 250	–	45.03	250.00	95	340	–	3.78
SF4	125.00	74.33	83.86	62.50	22.10	330	4	14.93
SF5	> 250	–	43.31	125	–	–	–	–
SF6	> 250	–	32.89	250	–	–	–	–
K001	62.50	41.23	76.89	250	88.38	697	16.91	7.88
K002	> 250	> 250	38.05	> 250	> 250	–	–	–
K003	125	74.33	82.05	31.25	18.58	180	2.42	9.69
K004	125	52.56	85.02	31.25	11.05	190	3.61	17.19
Ivermectin (μM)	5	3.05	5.80	2.5	1.57	250	81.96	159.23
DEC-C (μM)	1000	314.98	62.54	500	297.30	9000	28.57	30.27

^a^LC_100_ = 100 % reduction in motility indicates death of parasite

^b^IC_50_ = concentration of the agent at which 50 % inhibition in motility of the parasites is achieved

^c^CC_50_ = concentration at which 50 % of cells are killed

*Abbreviations*: *SI* Selectivity Index (CC_50_/IC_50_), *DEC-C* Diethylcarbamazine-citrate

Motility score of parasite in Control wells (parasite + DMSO only) = 4 (i.e. parasites were motile and highly active); MTT absorbance values (OD_510nm_) of control wells (parasites + DMSO only) = 0.64

### Evaluation of antifilarial efficacy *in vivo*

*Meriones unguiculatus* bearing intraperitoneally instilled adult worms and *M. coucha* inoculated with *B. malayi* L3 and showing microfilaremia in peritoneal cavity on day 3 post adult worm transplantation (p.a.t.) and progressive rise in microfilaraemia in peripheral circulation from day 120–180 post larval inoculation (p.l.i.), were employed as primary and secondary screening models, respectively, for assaying the antifilarial efficacy of the test agents.

The test agents were pulverised to fine powder and suspended in 1 % gum acacia/0.1 % Tween-80 in sterile distilled water. DEC-C and ivermectin were prepared in distilled water and ≤ 0.1 % Tween-80, respectively. The suspensions/solutions of the agents were prepared daily before administration to the animals.

In the *M. unguiculatis* model, the ethanolic extract (A001) was administered at 500 mg/kg, orally. Compounds K003 and K004 were given at 100 mg/kg body weight, subcutaneously (s.c.). The reference drugs DEC-C and ivermectin were administered to *M. unguiculatus* at 25 and 1 mg/kg body weight, respectively, while the *M. coucha* model received DEC-C at 50 mg/kg, body weight. The treatment was given for 5 consecutive days starting from day 1 to 5 through oral (A001) or s.c. (rest of the test compounds and reference drugs) routes. Infected animals treated with vehicle only were used as control.

Male *M. unguiculatus* 8–10 weeks old (40–45 g) transplanted intraperitoneally with freshly isolated adult worms from *B. malayi-*infected *M. unguiculatus* were used for primary screening of test agents [22]. On day 3 p.a.t., the peritoneal fluid was aspirated and checked for the presence of mf. The treatment was started on day 7/8 p.a.t. and continued for 5 consecutive days. On day 7/8 post-initiation of treatment (p.i.t) and thereafter at fortnightly intervals, the peritoneal fluid was examined under microscope to assess the condition of mf. All animals were killed on day 56 p.i.t. and parasites were collected from p.c., counted and examined for their viability and motility under microscope [10].

*Mastomys coucha* harbouring 5–7 month-old (L3 induced) *B. malayi* infection and showing progressive rise in microfilaraemia was used as secondary screening model [23]. Mf count was recorded in peripheral blood (10 μl tail blood) just before initiation of the treatment (day 0), on day 7/8 and thereafter at weekly intervals till day 91 p.i.t. The animals were killed on day 91 p.i.t.

As a standard practice (following an accepted protocol) all the *M. unguiculatus* and *M. coucha* treated with test agents and vehicle were observed daily for their general health and behaviour (cage side observations) throughout the course of treatment and thereafter till termination of the experiment.

Microfilaricidal efficacy of the test agents was evaluated at each time point p.i.t and expressed as percent change in mf count over the pretreatment level [6].

Macrofilaricidal/adulticidal efficacy of the agents was assessed by calculating percent reduction in adult worm recovery in treated animals over untreated animals [6]. Embryostatic/embryotoxic effect of agents was assessed by examining the uterine contents of female worms using the method described elsewhere [6] and the percent of sterilised worms was determined from total female worms recovered from the treated and control animals [6].

### Statistical analysis

Statistical analyses were carried out using GraphPad Prism 3.0 software. Results were expressed as the mean ± standard deviation (S.D.) of data obtained from 4–6 animals in two experiments. The data were analysed by Student’s ‘*t*’ test and one-way ANOVA followed by Tukey’s multiple comparison test, as appropriate. Differences with *P* < 0.05 were considered significant. The microfilaricidal efficacy of antifilarials was determined by comparing the slopes of control, K003 and DEC treated groups. The slopes of the regression line were compared by standard procedure.

## Results

### Structure elucidation of isolated molecules

**Fig. 3:**
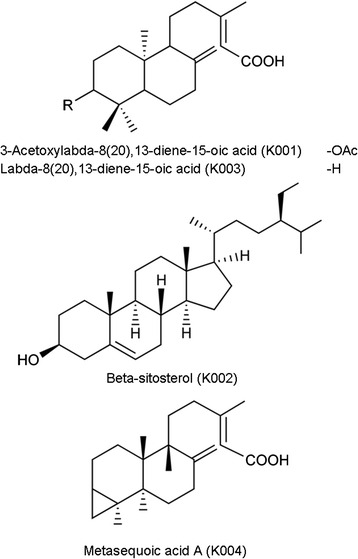
Structures of the compounds isolated from *Taxodium distichum*

The structures of the characterised molecules are given in Fig. 3.

***Compound 1 K001*** (3-Acetoxylabda-8(20),13-diene-15-oic acid: purity: > 95 %): white needles, molecular formula C_22_H_34_O_3_. ^1^H NMR (300 MHz, CDCl_3_): δ 1.7-1.8, 1.2-1.3 (H-1), 1.4-1.8 (H-2), 4.5 (H-3), 1.2 (H-5), 1.3-1.4 (H-6), 2.3-2.4, 2.0 (H-7), 1.5 (H-9), 1.6-1.8 (H-11), 2.2-2.4, 2.0 (H-12), 5.67 (H-14), 2.17 (H-16), 4.88, 4.53 (H-17), 0.88 (H-18), 0.85 (H-19), 0.72 (H-20), 2.06 (CH_3_CO) and ^13^C NMR (75 MHz, CDCl_3_): 37.1 (C-1), 24.6 (C-2), 81.0 (C-3), 38.4 (C-4), 55.0 (C-5), 24.1 (C-6), 38.3 (C-7), 147.7 (C-8), 56.1 (C-9), 39.6 (C-10), 22.0 (C-11), 40.3 (C-12), 163.9 (C-13), 115.4 (C-14), 172.4 (C-15), 19.6 (C-16), 107.3 (C-17), 28.6 (C-18), 16.9 (C-19), 14.9 (C-20), 21.6 (CH_3_CO), 171.3 (CH_3_CO). [M + H-H_2_O]^+^ 345.5847.

***Compound 2 K002*** (Beta-sitosterol, purity: > 97 %): white needles, molecular formula C_29_H_50_O. ^1^H NMR (300 MHz, CDCl_3_): δ1.8, 1.9 (H-1), 1.4, 1.9 (H-2), 3.5 (H-3), 2.3 (H-4), 5.35, 5.37 (H-6), 2.0, 1.85 (H-7), 1.5, 2.0 (H-8), 0.94 (H-9), 1.5 (H-11), 2.0-2.1 (H-12), 1.05 (H-14), 1.25, 1.6 (H-15), 1.8 (H-16), 1.08 (H-17), 0.69 (H-18), 0.94 (H-19), 1.35 (H-20), 0.85 (H-21), 1.3 (H-22), 1.56 (H-23), 0.9 (H-24), 1.7, 1.3,1.2 (H-25), 1.02 (H-26), 1.1 (H-27), 1.2 (H-28), 0.88 (H-29) and ^13^C NMR (75 MHz, CDCl_3_): 37.6 (C-1), 32.3 (C-2), 72.2 (C-3), 42.6 (C-4), 141.1 (C-5), 122.1 (C-6), 32.0 (C-7), 32.3 (C-8), 50.5 (C-9), 36.9 (C-10), 21.4 (C-11), 40.1 (C-12), 42.6 (C-13), 56.4 (C-14), 23.4 (C-15), 28.6 (C-16), 57.1 (C-17), 12.2 (C-18), 19.1 (C-19), 36.5 (C-20), 19.4 (C-21), 34.3 (C-22), 24.7 (C-23), 46.2 (C-24), 29.5 (C-25), 19.8 (C-26), 20.2 (C-27), 26.4 (C-28), 12.3 (C-29). [M + H]^+^ 414.3418.

***Compound 3 K003*** (labda-8(20),13-diene-15-oic acid: purity: > 95 %): Pale yellow oil, molecular formula C_20_H_32_O_2_. ^1^H NMR (300 MHz, C_6_D_6_): δ 1.4, 1.1 (H-1), 1.5-1.6 (H-2), 1.1, 1.2 (H-3), 0.9 (H-5), 1.4, 1.6 (H-6), 1.9-2.3 (H-7), 1.4 (H-9), 1.3-1.6 (H-11), 1.5-2.4, 2.0 (H-12), 5.86 (H-14), 2.2 (H-16), 4.85, 4.45 (H-17), 0.82 (H-18), 0.77 (H-19), 0.63 (H-20) and ^13^C NMR (75 MHz, C_6_D_6_): 38.7 (C-1), 19.8 (C-2), 42.5 (C-3), 33.8 (C-4), 55.7 (C-5), 24.8 (C-6), 39.2 (C-7), 148.3 (C-8), 56.6 (C-9), 40.0 (C-10), 21.9 (C-11), 40.4 (C-12), 164.1 (C-13), 115.6 (C-14), 173.1 (C-15), 19.4 (C-16), 106.9 (C-17), 33.8 (C-18), 22.0 (C-19), 14.7 (C-20). [M + H]^+^ 305.2917.

***Compound 4 K004*** (Metasequoic acid A: purity: > 95 %): Pale yellow oil, molecular formula C_20_H_30_O_2_. ^1^H NMR (300 MHz, CDCl_3_): δ 1.55 (H-1), 1.6 (H-2), 0.45 (H-3), 1.50-1.51 (H-5), 1.3, 1.9 (H-6), 2.0-2.4 (H-7), 1.53-1.54 (H-9), 0.8 (H-11), 2.0-2.4 (H-12), 5.68 (H-14), 2.17 (H-16), 4.89, 4.54 (H-17), 1.02 (H-18), 0.66 (H-19), 0.51 (H-20) and ^13^C NMR (75 MHz, CDCl_3_): 35.0 (C-1), 21.36 (C-2), 18.25 (C-3), 20.03 (C-4), 50.5 (C-5), 26.2 (C-6), 38.4 (C-7), 148.7 (C-8), 55.9 (C-9), 39.1 (C-10), 22.1 (C-11), 40.5 (C-12), 164.2 (C-13), 115.3 (C-14), 172.6 (C-15), 19.6 (C-16), 107.8 (C-17), 28.6 (C-18), 18.28 (C-19), 13.8 (C-20). [M + H]^+^ 303.2429.

The ^1^H and ^13^C NMR values for these compounds were similar to those reported in literature [25–29].

### *In vitro* activity of test agents on mf and adult worms

Results of *in vitro* activity of ethanolic extract (A001) of aerial parts of the plant, its fractions, subfractions and compounds are shown in Table 1*.* A001 was found to be more effective in killing mf (LC_100_: 3.91 μg/ml) than adult worms (LC_100_: 15.63 μg/ml) and the IC_50_ values for the respective parasite stages were found to be 1.95 and 10.00 μg/ml. A001 exerted 47 % inhibition in MTT-reduction assay over the DMSO control. Of the four fractions F001 was more effective in killing (100 % killing) mf (LC_100_: 7.83 μg/ml) requiring four times lesser concentration than needed for killing adult worms (LC_100_: 31.25 μg/ml); the fraction exerted 80 % inhibition in MTT reduction potential of the adult parasites. Of the six subfractions, SF2 killed both adult worms and mf (LC_100_: 7.83 μg/ml) and exerted 78 % inhibition in MTT-reduction assay. SF1 was also active but it was more microfilaricidal (LC_100_: 7.83 μg/ml) than macrofilaricidal (LC_100_: 31.25 μg/ml); the IC_50_ value of both the subfractions, SF1 and SF2 against mf was < 3.95 μg/ml. SF4 was less effective on motility of mf (LC_100_: 62.5 μg/ml) and adult worms (LC_100_: 125 μg/ml) but it caused 84 % inhibition in MTT reduction assay. The 2 diterpenoid compounds K003 and K004 isolated from SF2 killed mf (LC_100_: 31.25 μg/ml) and adult worms (LC_100_: 125 μg/ml); both compounds produced > 80 % inhibition in MTT-reduction assay.

DEC-C required 500 μM (mf) to 1000 μM (adult worms) to kill the parasite stages whereas ivermectin required much lower concentrations to kill both the parasite stages (LC_100_ for mf and adult worms were 2.5 μM and 5 μM, respectively).

In summary, compounds K003 and K004 of A001 were effective in killing mf and adult worms *in vitro.*

### *In vivo* activity of antifilarials: *B. malayi-M. unguiculatus* model

**Fig. 4:**
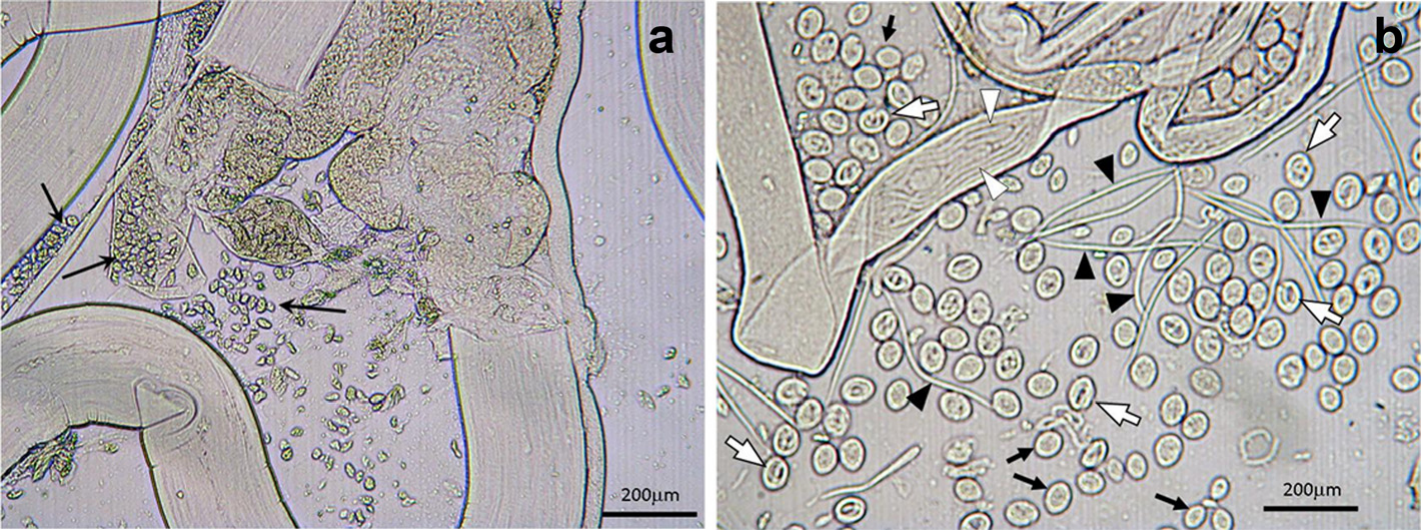
Embryostatic effect of compound K003 of *Taxodium distichum* in female parasites of *Brugia malayi* in *Meriones unguiculatus*. **a** Uterus of female worm contained distorted eggs and the embryos were devoid of developing microfilariae (mf) (*arrows*). **b** Female worm from vehicle treated animals (control) showing eggs (*arrows*), developing mf in embryos (*hollow arrows*) and fully developed mf (*hollow arrowheads*); fully developed mf expelled from uterus by gentle pressure on the worm during preparation was also seen around the worm (*filled arrowheads*)

Table 2 shows antifilarial efficacy of A001 and compounds K003 and K004 in *B. malayi*-*M. unguiculatus* model (primary screening model). A001 (500 mg/kg, orally) exhibited remarkable adulticidal activity; five of the six treated animals showed 100 % macrofilaricidal (*q* = 16.43, *P* < 0.001) activity while the sixth animal showed motile and active adult parasites and 28.57 % of the female parasites showed non-viable mf or dead/distorted embryonic stages in uterus. One-way ANOVA and Tukey’s multiple comparison tests showed that compound K003 (100 mg/kg) exerted remarkable embryostatic activity (> 95 %; *F*_(4,20)_ = 88.56, *P* < 0.001; Table 2; Fig. 4a) as compared to K004, ivermectin, DEC and vehicle treated animals (Table 2). At 100 mg/kg dose, both the purified compounds produced > 25 % macrofilaricidal activity (K003: 27 %; K004: 32.20 %; Table 2). However, K003 and K004 treated animals showed motile and active mf in the p.c. and this was not much different from the status of mf in p.c. of the control animals (data not shown) indicating that the compounds were ineffective against mf in the *M. unguiculatus* model.

**Table 2:** Antifilarial activity of crude extract of aerial parts of *Taxodium distichum*, its molecules, and the reference drugs ivermectin and DEC against *Brugia malayi* in jirds, *Meriones unguiculatus* (mean ± standard deviation)

Antifilarial agent	Dose mg/kg s.c. × 5 days (*n*)	Worms recovered			Sterilised female worm count (%)
		Male	Female	Total (% reduction over untreated)	
A001 (Crude extract)	500 p.o. (6)	0	0	0*** (5)	–2 (28.57)
				11.00 (1)^b^	
K003	100 (5)	0.40 ± 0.89	8.00 ± 0.71	8.40 ± 1.14	8.00 ± 0.71
				(27.12)	(> 95)***
K004	100 (5)	0	8.00 ± 0.82	8.00 ± 0.82	1.50 ± 0.58
				(32.20)	(18.85 ± 7.22)
Ivermectin^a^	1 (4)	2.75 ± 0.96	6.25 ± 1.50	9.67 ± 2.52	2.75 ± 0.96
				(22.43)	(44.29 ± 5.15)**
DEC-C^a^	25 (6)	2.60 ± 0.55	6.60 ± 1.14	9.20 ± 1.48	0.60 ± 0.89
				(22.03)	(7.86 ± 11.41)
Control (Vehicle treated)	– (5)	3.40 ± 1.97	8.40 ± 2.07	11.80 ± 0.45	0.40 ± 0.55
					(5.71 ± 7.82)

^a^Reference drugs; DEC-C: Diethylcarbamazine-Citrate

^b^One animal showed presence of live adult worms but not dead/calcified worms

**P < 0.01 (ivermectin *vs* Control/DEC/K004); ***P < 0.001 (K003 *vs* control/K004/ivermectin/DEC; A001 *vs* control/K003/K004/ivermectin/DEC)

The reference drug ivermectin did not affect mf in p.c. (data not shown) but produced mild but not statistically significant effect on adult worms. The drug also produced significant embryostatic effect (44 %; *t* = 18.92, *P* < 0.01; Table 2) and one-way ANOVA followed by Tukey’s multiple comparison test revealed that the embryostatic effect produced by ivermectin was significantly greater (*F*_(1,8)_ =358.04, *P* < 0.01; Table 2) than that seen in DEC/K004 treated or untreated animals.

In the *M. unguiculatus* model, DEC did not affect mf count or female reproductive potential but exerted mild macrofilaricidal activity (Table 2). Female worms from control *M. unguiculatus* showed normal and healthy uterine contents (Fig. 4b). Cage-side observations did not reveal any abnormalities in the general health and behavior of *M. unguiculatus* of any group at any time point of observation period. In summary, in the *M. unguiculatus* model A001 killed all the adult worms in > 80 % of infected animals and K003 exhibited > 95 % embryostatic activity.

### *In vivo* activity of antifilarials: *Brugia malayi-M. coucha* model

**Fig. 5:**
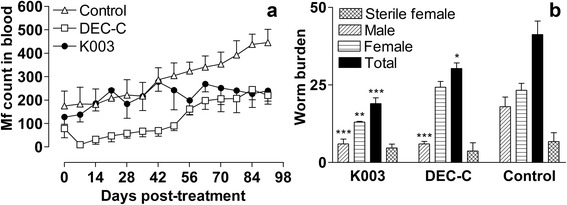
Antifilarial activity of compound K003 of *Taxodium distichum* and the reference drug diethylcarbamazine-citrate (DEC-C) against *Brugia malayi* in *Mastomys coucha*. Mf count values are mean ± standard deviation of 4–6 animals in two experiments. **a** Pre- and post- treatment levels of microfilaraemia in treated and control animals. Statistical significance levels are given in Table 3. **b** Adult worms and sterile female worms recovered from control, K003 and DEC treated animals

Antifilarial efficacy of K003 and reference drug DEC in *B. malayi*-*M. coucha* system (secondary screening model) is presented in Fig. 5. K003 treatment had more or less no effect on microfilaraemia till day 42 p.i.t., but thereafter microfilaraemia stopped increasing as compared to control (Fig. 5a) (i.e. there was no statistically significant rise in microfilaraemia after day 42 p.i.t) and by day 80 p.i.t, the mf levels were significantly lower than those in untreated control (*t* = 4.82, *P* = 0.0004) and equaled the levels in DEC treated group (*t* = 0.062, *P* = 0.9516). The trend in post-treatment microfilaraemia (Fig. 5a) was ascertained by simple regression equation analysis (Table 3) which showed that all the lines have significant slopes and the rate of change of microfilaraemia between any two treatment groups (i.e, control vs K003, control vs DEC, and DEC vs K003) was statistically significant. K003 also exerted 53.94 % macrofilaricidal (*q* = 7.557, *P* < 0.001; Fig. 5b) activity compared to untreated control animals; recovery of female parasites from the treated animals was lower than that of control animals (*q* = 10.20, *P* < 0.0001). More than 36 % of the surviving female parasites were found sterile although this was not statistically significant (*P* > 0.05) when compared with the sterile female parasites of control animals (*q* = 0.87, *P* = 0.913). Further, one-way ANOVA analysis revealed that K003 was superior to DEC with respect to macrofilaricidal efficacy as evidenced by lesser worm recovery from K003 treated animals (*q* = 4.08, *P* = 0.041).

**Table 3:** Regression equations determining the trends in microfilarial counts in Fig. 5a

Antifilarial agent	Regression line	*P*-value
K003	183.65 + 0.82 days	0.04
	(0.352)	
DEC-C	-21.04 + 2.95 days	< 0.001
	(0.24)	
Untreated control	135.44 + 3.350 days	< 0.001
	(0.170)	

Values in parentheses represent standard errors of slopes

DEC-C (50 mg/kg) which is chiefly a microfilaricide, caused > 80 % reduction in microfilarial count on day 7/8 p.i.t. which gradually increased and relapsed by day 49 p.i.t.; the count further increased rapidly and crossed the pretreatment level by day 56 p.i.t. (Fig. 5a). DEC also showed around 26.47 % macrofilaricidal and 20 % female sterilising activity but this activity was not statistically significant compared to control (Fig. 5b); however, significantly lower numbers of male parasites were recovered from DEC treated animals (*q* = 7.28, *P* = 00074) than from the control animals.

Microfilaraemia in control animals was progressively higher than the pretreatment level and never equalled the 0 day level. About 28 % of female worms recovered from the control animals were found to be sterilised. None of the animals showed any signs of toxicity as inferred from cage-side observations of gross clinical signs. In summary, in *B. malayi*-*M. coucha* model, K003 produced significant macrofilaricidal activity and rendered more than 36 % female worms sterile; it also stopped any further rise in microfilaraemia after day 42 p.i.t.

## Discussion

In the present bioassay-guided fractionation of the ethanolic extract from the aerial parts of the conifer *T. distichum*, we found that the antifilarial activity was localised to labdane diterpenoids K003 and K004 of the subfractions SF2. Although there are reports that diterpenoids show antibacterial, antifungal, anti-protozoal and anti-inflammatory [30, 31] activities, there are no previous reports on antifilarial activity of the compounds from the conifer *T. distichum*.

In line with our earlier studies on a number of plant products [5, 9, 11], the main emphasis of the present study was to identify antifilarial compounds of *T. distichum* that preferentially target the adult worms and embryogenesis in the female worms. This is considered as a superior approach for control of filariasis (WHO recommends this approach for new drug discovery) since killing/interfering with embryogenesis of even one female adult worm will stop production of millions of mf that circulate in the blood and this may effectively interrupt or reduce the transmission of the infection. Unfortunately, the currently available antifilarial drugs are largely microfilaricides with little effect, if any, on adult worms.

In the present study, we found micro- and macrofilaricidal activity in the crude extract and purified compounds K003 and K004 *in vitro*. However, the IC_50_ of pure compounds was higher than that of crude extract. At present the reasons for this differences in the activity between ethanolic extract and the compound isolated from it are not clear, but it appears that greater activity in the crude extract could be due to additive or synergistic effects of K003 and K004 in it; other substance(s) such as flavonoids in the ethanolic extract might have also contributed to this activity since we have recently shown that some flavonoids possess antifilarial activity [22]. In the *M. unguiculatus* model (primary model), the ethanolic extract (A001) showed a remarkable macrofilaricidal activity while the purified compound K003 of the extract exerted embryostatic activity only with mild macrofilaricidal activity. Conversely, in the *M. coucha* model (secondary model), the compound exhibited significant macrofilaricidal activity but only mild (statistically not significant) embryostatic activity. However, *M. coucha* model exhibits close similarity to human infection in several respects such as the course of infection [19], pathogenesis of disease manifestations [32, 33], immune responses [34] and responses to antifilarials [35–38]. The differences in the response of the two rodent models to the same compound are apparently due to differences in the way they metabolise the compound and the resultant variation in the ratio of bioactive metabolites responsible for macrofilaricidal and embryostatic effects.

In the *M. coucha* model, microfilaraemia stopped increasing after day 42 p.i.t. with K003, and by day 80 p.i.t., the levels were significantly lower than those in untreated control and equaled the levels in DEC treated group. Summing it up, the overall microfilaraemia was the highest in control group and the least in DEC group with K003 taking the intermediate position (Fig. 5a). The reasons for lesser changes in microfilaraemia in K003-treated group are not clear at present. But it appears that late suppression of peripheral microfilaraemia in *M. coucha* is due to the adult worm mortality and the inhibitory effect on embryogenesis produced by K003. As the adult worm mortality was higher in males than in females, this may have lead to lower mating rate and consequent irregular mf production. With respect to the standard reference drugs, both DEC and ivermectin were ineffective against mf in *M. unguiculatus* model but DEC showed strong microfilaricidal effect in *M. coucha* [9–11, 35–37, 39, 40]. This is in agreement with the known microfilaricidal efficacy of DEC in *M. coucha*, cats [41] and humans [42–44]. Similar differences between *B. malayi*-*M. unguiculatus* and *B. malayi-M. coucha* systems with respect to antifilarial activity of DEC and ivermectin have been reported by several investigators [6, 36–38]. In a similar vein, DEC showed mild adulticidal and embryostatic activities in the *M. coucha* model and poor adulticidal activity in the *M. unguiculatus* model. In the *M. unguiculatus* model, K003 and ivermectin showed strong and moderate embryostatic effect, respectively, while DEC was ineffective.

The mechanism by which the labdane diterpenoid compounds K003 and K004 of *T. distichum* exerted filaricidal activity *in vitro* and *in vivo* remains to be investigated. Several possible mechanisms may be considered including an indirect action *via* the known antibacterial activity of diterpenoids of this plant. The antibacterial activity might have resulted in killing of the filarial symbionts *Wolbachia* spp. which supports the survival of the parasite [45].

## Conclusions

Ethanolic extract A001 prepared from aerial parts of the plant *T. distichum* was solvent fractionated, subfractionated and four molecules: 3-Acetoxylabda-8(20), 13-diene-15-oic acid (K001), Beta-sitosterol (K002), labda-8(20),13-diene-15-oic acid (K003) and Metasequoic acid A (K004) were isolated. The structures of the compounds were elucidated by spectroscopic analysis. A001 and labdane diterpenoid compounds K003 and K004 showed micro- and macrofilaricidal activity *in vitro*. In the *M. unguiculatus* model, A001 killed all the adult worms in > 80 % of infected animals; the active principle was localised to labdane diterpenoid compound K003 which showed remarkable embryostatic activity in this model. In *M. coucha* model the compound K003 exerted significant macrofilaricidal activity with late suppression of peripheral microfilaraemia and some embryostatic activity. Thus, in conclusion the findings indicate that labdane diterpenoid molecule(s) may provide valuable lead for design and development of new macrofilaricidal agent(s). Future studies will include chemical modification of K003 and K004 to maximise the antifilarial activity; regulatory toxicity studies will be carried out on the molecules maximised for activity.
