# Erratum to: Antifilarial activity of diterpenoids from *Taxodium distichum*

**DOI:** 10.1186/s13071-017-2119-3

**Published:** 2017-04-26

**Authors:** Vikas Kushwaha, Kirti Saxena, Richa Verma, Shiv K. Verma, Deepali Katoch, Neeraj Kumar, Brij Lal, P. Kalpana Murthy, Bikram Singh

**Affiliations:** 1 Division of Parasitology, CSIR-Central Drug Research Institute, New Campus, BS 10/1, Sector 10, Jankipuram Extension, Lucknow, 226 031 India; 2 Natural Product Chemistry and Process Development Division, CSIR-Institute of Himalayan Bioresource Technology, Palampur, 176 061 HP India; 3 Biodiversity Division, CSIR-Institute of Himalayan Bioresource Technology, Palampur, 176 061 HP India; 4 Present Address: USDA, ARS, APDL, BARC-East Bldg 1001, 10300 Baltimore Avenue, Beltsville, MD 20705 USA

## Erratum

In the paper [1] the present address of the Corresponding Author ‘P. Kalpana Murthy’ should read as:

CSIR Emeritus Scientist, Department of Zoology, Lucknow University, University Road, Lucknow 226007, India.

The following information is added in the ‘**Acknowledgements**’ section:

“Thanks are due to CSIR, New Delhi, for award of Emeritus Scientist Project to PKM. Thanks are also due to Vice Chancellor, Lucknow University, Lucknow, for providing laboratory space and computational facilities to PKM for carrying out part of the study, data analysis and manuscript preparation.”

## References

[1] Kushwaha V, Saxena K, Verma R, Verma SK, Katoch D, Kumar N (2016). Antifilarial activity of diterpenoids from *Taxodium distichum*. Parasit Vectors.

